# 3D Printing of Flexible Mechanical Metamaterials: Synergistic Design of Process and Geometric Parameters

**DOI:** 10.3390/polym15234523

**Published:** 2023-11-24

**Authors:** Nan Li, Chenhao Xue, Shenggui Chen, Wurikaixi Aiyiti, Sadaf Bashir Khan, Jiahua Liang, Jianping Zhou, Bingheng Lu

**Affiliations:** 1 School of Mechanical Engineering, Xinjiang University, Xinjiang, Urumqi 830047, China; dglgln@163.com (N.L.); xch1107@stu.xju.edu.cn (C.X.); dgutchensg@163.com (S.C.); linkzhou@163.com (J.Z.); bhlu@mail.xjtu.edu.cn (B.L.); 2 School of Education (Normal School), Dongguan University of Technology, Dongguan 523808, China; 3 School of Art and Design, Guangzhou Panyu Polytechnic, Guangzhou 511483, China; 4 School of Manufacturing Science and Engineering, Key Laboratory of Testing Technology for Manufacturing Process, Ministry of Education, Southwest University of Science and Technology, Mianyang 621010, China; sadafbashirkhan@swust.edu.cn; 5 Dongguan Institute of Science and Technology Innovation, Dongguan University of Technology, Dongguan 523808, China; jiahua_liang1114@163.com

**Keywords:** mechanical metamaterials, TPMS structures, gradient architecture, selective laser sintering (SLS), thermoplastic polyurethane (TPU)

## Abstract

Mechanical metamaterials with ultralight and ultrastrong mechanical properties are extensively employed in various industrial sectors, with three-periodic minimal surface (TPMS) structures gaining significant research attention due to their symmetry, equation-driven characteristics, and exceptional mechanical properties. Compared to traditional lattice structures, TPMS structures exhibit superior mechanical performance. The mechanical properties of TPMS structures depend on the base material, structural porosity (volume fraction), and wall thickness. Hard rigid lattice structures such as Gyroid, diamond, and primitive exhibit outstanding performance in terms of elastic modulus, energy absorption, heat dissipation, and heat transfer. Flexible TPMS lattice structures, on the other hand, offer higher elasticity and recoverable large deformations, drawing attention for use in applications such as seat cushions and helmet impact-absorbing layers. Conventional fabrication methods often fail to guarantee the quality of TPMS structure samples, and additive manufacturing technology provides a new avenue. Selective laser sintering (SLS) has successfully been used to process various materials. However, due to the layer-by-layer manufacturing process, it cannot eliminate the anisotropy caused by interlayer bonding, which impacts the mechanical properties of 3D-printed parts. This paper introduces a process data-driven optimization design approach for TPMS structure geometry by adjusting volume fraction gradients to overcome the elastic anisotropy of 3D-printed isotropic lattice structures. Experimental validation and analysis are conducted using TPMS structures fabricated using TPU material via SLS. Furthermore, the advantages of volume fraction gradient-designed TPMS structures in functions such as energy absorption and heat dissipation are explored.

## 1. Introduction

Mechanical metamaterials, as a crucial subfield of metamaterials, aim to achieve extraordinary mechanical properties that conventional materials cannot provide [1]. These properties often include unique zero or negative mechanical parameters, such as Poisson’s ratio and elastic modulus [2]. Mechanical metamaterials have demonstrated their lightweight nature and outstanding energy absorption capabilities in various research areas, including sports equipment, medical rehabilitation, biomedical sciences, and aerospace [3]. As technology and industry rapidly advance, there is an increasing demand for mechanical metamaterials with exceptional mechanical properties in a wide range of industrial sectors, including aerospace, mechanical engineering, and civil engineering [4]. These materials are sought after for their applications in energy absorption, highlighting their importance in addressing industry-specific needs [5].

In the pursuit of achieving ultralight and super-strong mechanical properties, various lattice structures inspired by nature and topology have been designed as mechanical metamaterials, which can be divided into ordered arrangements (such as honeycomb structures) and disordered arrangements (such as Voronoi diagram) according to their account; they can also be divided into truss/rod-like metamaterials (such as face-centered cubic, body-centered cubic) and planar/thin-walled metamaterials (such as three-period minimal surfaces) according to their geometry. These metamaterials are widely used in modern industrial fields [6]. Among these, three-periodic minimal surface (TPMS) structures have gained significant research attention due to their symmetry, equation-driven characteristics, and outstanding mechanical performance [7,8]. When compared to traditional truss lattice structures, TPMS structures exhibit superior mechanical properties. The mechanical performance of TPMS structures is influenced by the properties of the base material, the structural porosity (volume fraction), and the wall thickness. In recent years, a significant portion of research has been focused on rigid lattice structures, such as Gyroid, diamond, and primitive structures, manufactured from materials such as polymers (e.g., PA12) and metals (e.g., Ti-6Al-4 V), with their mechanical properties indirectly controlled via the variation of geometric parameters [9]. These structures showcase exceptional performance in terms of elastic modulus, energy absorption, heat dissipation, and thermal exchange, among other aspects [10]. On the other hand, flexible TPMS lattice structures have emerged as a recent and growing research area [11]. In contrast to the advantages of rigid lattice structures in load-bearing supports, flexible TPMS lattice structures offer higher elasticity and recoverable large deformations, making them particularly relevant in applications such as seat cushions and helmet impact-absorbing layers [1,12,13]. Last, in the engineering applications of lattice structures, optimization is needed to tailor these structures to complex operating conditions based on functional requirements. Research has shown that, for rigid materials, structures with gradient rigidity and new lattice structures resulting from the combination of two lattice types can enhance the energy absorption efficiency compared to that of uniform lattice structures [14,15,16].

In the field of mathematics, a minimal surface refers to a surface with an average curvature of zero. Therefore, we can obtain geometric models of TPMS (three-periodic minimal surface) structures with smooth and continuous surfaces using mathematical methods. Several common TPMS structures include Gyroid, diamond, and Schwarz’P, and they can be obtained according to different mathematical models. However, due to the geometric characteristics of TPMS structures, traditional fabrication methods cannot guarantee the quality of the samples. With the development of additive manufacturing technology, a new approach has been provided for the fabrication of TPMS structures [10,17,18,19]. Additive manufacturing (AM) makes it possible to produce these complex TPMS structures. For example, in recent years, the classical additive processes FDM and SLA/DLP have been used to prepare metamaterials. The FDM process has a wide range of materials (such as PLA, ABS, PA) and low cost. SLA has the characteristic of high-precision molding, which is an ideal process for biological scaffolds. Still, these processes also have some limitations in the preparation of metamaterials, such as the need for adding support and a relatively slow molding speed [20]. Selective laser sintering (SLS), as one of the most commercially used additive manufacturing methods, has successfully processed materials including hard polymers from the PA series, PP series, and flexible polymers such as the TPU series [21,22,23,24]. The development of SLS allows researchers to design and produce TPMS structures without the need for supports. However, due to the constraints of the layer-by-layer manufacturing process, it is impossible to avoid the anisotropy introduced by interlayer bonding [25,26]. In other words, for an isotropic structure, the mechanical performance of 3D-printed parts is always dependent on the build direction [27,28,29,30]. Recent research has shown that common 3D-printing processes can exhibit directional effects on the mechanical performance of test parts due to the influence of the anisotropy introduced by layer-by-layer manufacturing. David W. and his team, for instance, applied compressive loads in both the vertical and horizontal directions to 3D-printed Gyroid structures [31]. The force-displacement curves indicated that the anisotropy induced by the layer orientation in 3D printing affected the Gyroid structure, which had previously been theoretically approximated as nearly isotropic [1,15,30,32].

In this work, we propose a process data-driven geometric parameter optimization design method for TPMS structures. By adjusting the gradient of the volume fraction, the method strives to overcome the influence of elastic anisotropy caused by the layer orientation of the 3D-printed (selective laser sintering) isotropic lattice structure while keeping the quality of the lattice structure unchanged. To better analyze the influence of construction direction on the volume fraction gradient design, we selected SLS-shaped TPU material for preparing the TPMS structure. This is because flexible materials have higher elasticity than hard materials and can withstand greater deformation. In addition, the theoretical elastic properties of most TPMS structures are nearly isotropic. Finally, we further discuss the advantages of the TPMS structure with a volume fraction gradient design compared with the homogeneous TPMS structure in terms of energy absorption and heat dissipation.

## 2. Materials and Methods

### 2.1. Parameterized Structural Design

**Figure 1:**
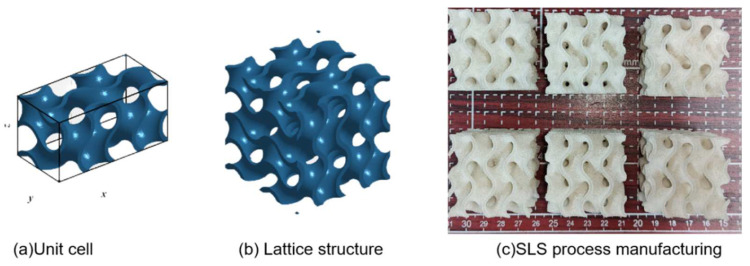
3D-printing model and sample. (**a**) Lattice structure unit cell model. (**b**) Lattice structure model. (**c**) Lattice structure sample preparation.

In this work, a series of TPMS geometries based on chip gyroscopes are studied, and the function of the Gyroid (U_G_) structure is defined as:

(1) $$U_{G}\left(x,y,z\right)=Sin\left(x\right)Cos\left(y\right)+Sin\left(y\right)Cos\left(z\right)+Sin\left(z\right)Cos\left(x\right)-T$$

where x, y, and z are the three-dimensional dimensions and arrangement of the control lattice structural units [33], as shown in Figure 1a, and T is an arbitrary parameter used to control the curvature of the TPMS, thereby indirectly affecting the volume fraction (wall thickness) of the lattice structure, where the definition of the volume fraction V is as follows:

(2) $$V=\frac{V_{solid}}{V_{solid}+V_{void}}$$

The volumes of the solid and void in a lattice structure are represented by V_solid_ and V_void_, respectively. Simultaneously, when the fixed value T in the TPMS structural function equation is replaced by a new function T(x,y,z) [33], a gradient change in the volume fraction in a specified direction within the same lattice structure arrangement can be achieved.

### 2.2. Synergistic Design of the Process and Geometric Parameters

We propose a data-driven design method to address the anisotropic effects induced by 3D-printing processes on structural performance. This method allows for the optimization of elastic anisotropy in the X, Y, and Z directions of 3D-printed lattice structures without altering the lattice structure’s quality and volume via the design of volume fraction gradients within the lattice structure. Using this approach, better control and optimization of the performance of 3D-printed structures can be achieved to meet the requirements of various application scenarios.

First, a mathematical relationship F(T, V) between the volume fraction V and fixed curvature T is obtained using linear interpolation fitting of the implicit function equation of the TPMS with uniform volume fractions. Subsequently, the nonlinear mathematical relationship F(V, E) between the volume fraction V and the elastic modulus E of the TPMS structure is derived via numerical analysis simulations and experimental verification. Using this approach, parametrically controllable TPMS lattice structures can be achieved. Next, we define the anisotropic elasticity in the x, y, and z directions of lattice structures with a uniform volume fraction V_1_ obtained via experimental measurements in 3D printing.

(3) $$△E=\frac{2E_{z}-\left(E_{x}+E_{y}\right)}{2}$$

The mathematical model’s objective is to evaluate and quantify the anisotropy of the elastic modulus in the x, y, and z directions of a 3D-printed lattice structure under specified 3D process parameters and methods. In this context, the z direction represents the build direction in 3D printing, and the elastic modulus is denoted as E. To control the anisotropy of the elastic modulus of the 3D-printed lattice structure, the range of gradient variation in volume fraction, ΔV, also needs to be determined as mathematically defined below:

(4) $$△E=\frac{\left|F\left(V_{1},E\right)-F\left(V_{1}+△V,E\right)\right|}{2}$$

From the aforementioned mathematical model, we can obtain an accurate value of ΔV. However, this method not only requires finding the inverse function of F(V, E) but also involves a significant computational workload. Existing research has already demonstrated the existence of a nonlinear mathematical relationship between the volume fraction of the lattice structure and the elastic modulus [14]. Therefore, we can simplify as follows: First, calculate the first derivative of F(V, E) at V = V_1_. This can indicate the local change trend of the elastic modulus around the volume fraction V_1_. Based on this, ΔV is defined as follows:

(5) $$△V=\frac{△E}{F_{V=V_{1}}^{\prime }\left(V,E\right)}$$

The optimized geometric model for the construction direction of the lattice structure (in the z direction) is designed using a gradient approach, as shown in Figure 1, where the maximum volume fraction is represented as V1 + △V, and the minimum volume fraction is V1 − △V. In order to ensure that the mass remains unchanged before and after control, as shown in Equation 2, it is only necessary to keep the mean volume fraction of the geometric structure equal before and after control when V_solid_ + V_void_ is unchanged. What needs to be specifically noted is that △V is a parameter dependent on V_1_, and thus the control of elastic anisotropy in 3D-printed lattice structures is also centered around the fixed volume fraction lattice structure. Then, the mapping curvature value T ± ΔT can be determined using the equation F(T, V), and then the gradient function T(x,y,z) can be constructed using the first function through two points (T − ΔT, l) and (T + ΔT, l), the independent variable is the direction of the gradient, the other variables are constants, and l is the gradient length. The gradient range of the gradient structure can be determined using the above method.

### 2.3. Experiment and Simulation

In this study, a TPM3D P360 selective laser sintering (SLS) device was used for sample preparation (Figure 1). The sample is made of thermoplastic polyurethane (TPU), a flexible material, and its process parameters are shown in Table 1. After testing, it was found that when the unit size was greater than 10 mm, a volume fraction between 15% and 30% was more conducive to forming. Therefore, we select a uniform Gyroid lattice structure with a cell size of 20 mm, period k of 2 × 2 × 2, and volume fraction of 20% as the reference group. At the same time, the average volume fraction of the gradient TPMS lattice structure is 20% after optimizing the geometric parameters. Three samples of uniform structure and gradient structure were prepared, respectively. Table 2 shows the specific characterization of print quality.

The compression test of the sample prepared using selective laser sintering was carried out using an LD23.104 10 kN microcomputer-controlled electronic universal testing machine (China Force Test Scientific Instrument Co., Ltd., Hong Kong, China) to evaluate its mechanical properties. Before testing, the test sample was placed at 23 °C and 50% relative humidity for at least 16 h. When the total compressive strain is 50%, the compression experiment is carried out at a strain rate of 5 mm/min, and the corresponding compressive force-displacement data are recorded. At the same time, the data are recorded when the strain is 5%, and the ratio of stress-strain is calculated to obtain the experimental elastic modulus.

In this paper, the numerical homogenization method is used to evaluate the elastic modulus. The Gyroid unit was selected as the representative volume unit (RVE). According to the generalized Hooke’s law, the stress-strain relationship can be expressed as:

(6) $$\left\{\sigma \}=\left[C\right]\cdot \{\varepsilon \right\}$$

Among them, stress {σ} and strain {ε} are further described as:

(7) $$\{\sigma \}=\{\sigma_{11},\sigma_{22},\sigma_{33},\sigma_{23},\sigma_{31},\sigma_{12}{\}}^{T}$$

(8) $$\{\varepsilon \}=\{\varepsilon_{11},\varepsilon_{22},\varepsilon_{33},\varepsilon_{23},\varepsilon_{31},\varepsilon_{12}{\}}^{T}$$

Therefore, the stiffness matrix [C] is expressed as:

(9) $$[C]=\left[\begin{array}{cccccc} C_{11} & C_{12} & C_{13} & C_{14} & C_{15} & C_{16}\\ C_{21} & C_{22} & C_{23} & C_{24} & C_{25} & C_{26}\\ C_{31} & C_{32} & C_{33} & C_{34} & C_{35} & C_{36}\\ C_{41} & C_{42} & C_{43} & C_{44} & C_{45} & C_{46}\\ C_{51} & C_{52} & C_{53} & C_{54} & C_{55} & C_{56}\\ C_{61} & C_{62} & C_{63} & C_{64} & C_{65} & C_{66} \end{array}\right]$$

In addition, the TPMS structure is a cubic symmetric system with three independent constants, specifically, C_11_ = C_22_ = C_33_, C_12_ = C_13_ = C_23_, and C_44_ = C_55_ = C_66_. The remaining constants are zero. The stiffness matrix of Gyroid [C] can be simplified as follows: To calculate the values of C_11_, C_12_, and C_44_, at each step, one of the strains is 1, and the others are 0. Using this numerical homogenization method, the normal strain and shear strain are realized using finite element analysis to calculate the corresponding stress. For normal strain ɛ_11_ = 1, the boundary condition is set to:

(10) $$\left\{\begin{array}{c} \Delta l_{x}{|}_{x=l_{x}}=0.001l_{x}\\ \Delta l_{x}{|}_{x=0}=\Delta l_{y}{|}_{y=l_{y}}=\Delta l_{y}{|}_{y=0}=\Delta l_{z}{|}_{z=l_{z}}=\Delta l_{z}{|}_{z=0}=0 \end{array}\right.$$

For shear strain ɛ_31_ = 1, the boundary condition is set to:

(11) $$\left\{\begin{array}{c} \Delta l_{x}{|}_{z=l_{x}}=0.0005l_{z},\Delta l_{z}{|}_{x=l_{z}}=0.0005l_{x}\\ \Delta l_{z}{|}_{x=0}=\Delta l_{y}{|}_{y=l_{y}}=\Delta l_{y}{|}_{y=0}=\Delta l_{z}{|}_{z=l_{z}}=\Delta l_{x}{|}_{z=0}=0 \end{array}\right.$$

Finally, the stiffness tensor C can be used as the volume average result of the total stress, which can be described as:

(12) $$C_{ij}=\bar{\sigma }=\frac{1}{V}\int_{V}\sigma_{ij}dV$$

According to the stiffness matrix [C], the elastic modulus and anisotropy properties can be obtained simultaneously. The elastic modulus is calculated as follows:

(13) $$E=\frac{C_{11}^{2}+C_{12}C_{11}-2C_{12}^{2}}{C_{11}+C_{12}}$$

The Zener anisotropy index is a common method for evaluating anisotropic properties. If the structure is isotropic, the Zener anisotropy index will be 1. The Zener anisotropy index is calculated as follows:

(14) $$A=\frac{2C_{44}}{C_{11}-C_{12}}$$

**Figure 2:**
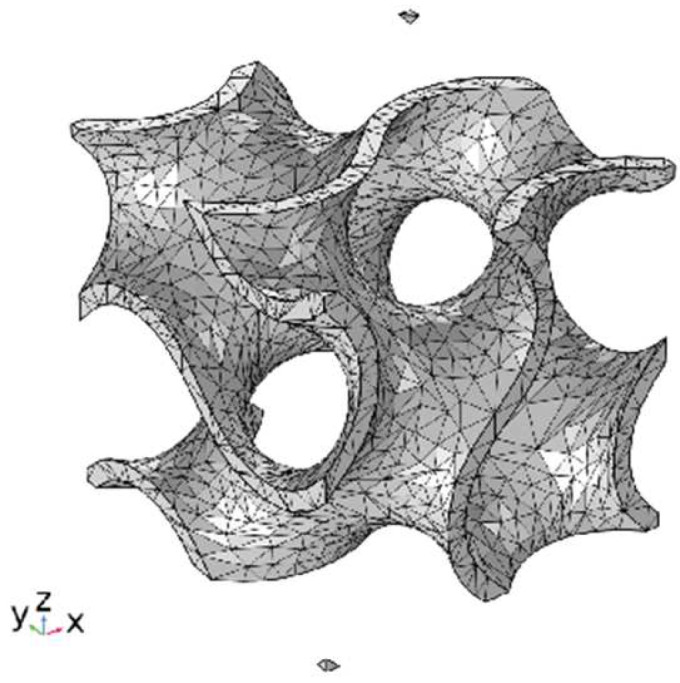
Structural finite element model.

**Figure 3:**
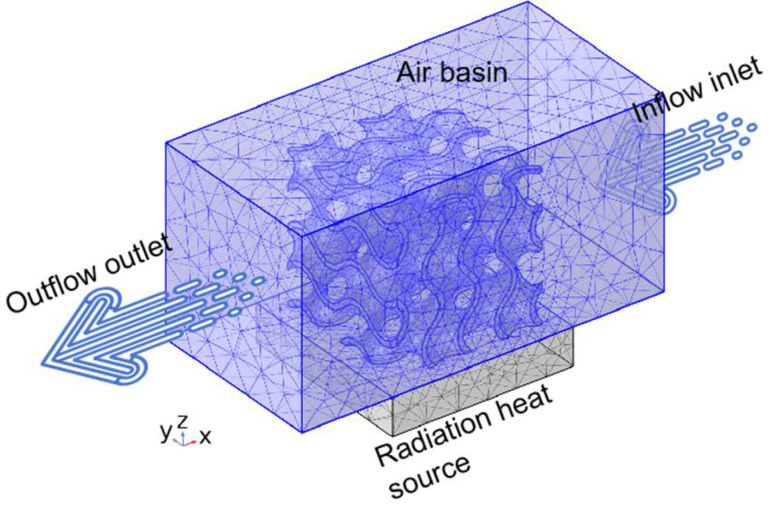
Finite element heat dissipation model.

In the finite element analysis of Gyroid structures with different volume fractions to calculate normal stress and shear stress, as shown in Figure 2, the finite element model adopts a free tetrahedral mesh, the boundary conditions are fixed and specified displacement (Equations (10) and (11)), and the elastic modulus and Poisson’s ratio are 14 MPa and 0.48. This is the average value of TPU samples prepared using SLS. At the same time, we also simulate the functional advantages of a gradient structure in heat conduction, as shown in Figure 3. The model adopted a tetrahedral mesh, the thermal conductivity of TPU was 0.5 W/(m × K) (refer to the internal material parameters of COMSOL), a heat source was placed under the uniform Gyroid structure and the gradient Gyroid structure, and the heat consumption rate of the heat source was 0.5 W. The surface radiation emissivity to the thermal environment is 0.8, which simulates the flow rate of 10 cm/s of 293.75 K air at room temperature.

## 3. Results

### 3.1. Influence of Interlayer Bonding on Mechanical Properties

**Figure 4:**
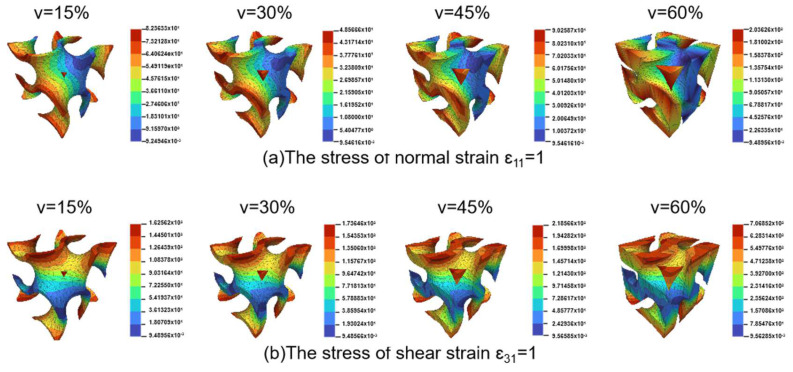
FEA results of normal and shear strain (unit: Kpa).

**Figure 5:**
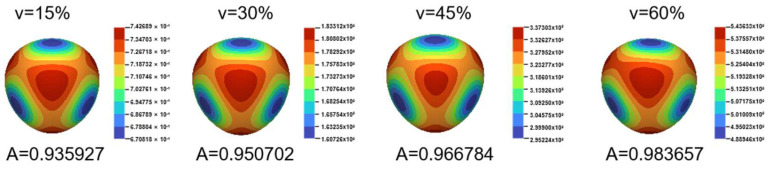
The homogenization theory simulates the results of three-dimensional elastic modulus surface (unit: MPa).

**Figure 6:**
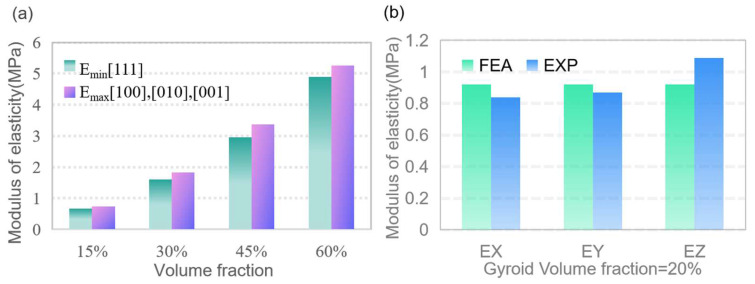
The elastic modulus of the uniform Gyroid structure. (**a**) The theoretical simulation value of homogenization. (**b**) The sample preparation test value of SLS process.

In this paper, COMSOL was used for finite element analysis, and the stress distribution shown in Figure 3 was obtained according to the boundary conditions. The volume fraction of Gyroid was set to 15%, 30%, 45%, and 60%, and the influence of the volume fraction on its performance was analyzed. The results of Figure 4 show that the stress distribution of Gyroid is similar for different volume fractions, the internal stress distribution is more uniform, and the stress concentration occurs at the outer sharp edges or thin walls. Based on the FEA results of positive strain and shear strain, the stiffness matrix of the Gyroid is obtained accurately. By drawing a 3D surface plot (Figure 5), we can observe how the elastic modulus in different directions behaves in the Gyroid structure with different volume fractions. First, the elastic modulus surface of the Gyroid structure is very close to that of a sphere, indicating that it has approximately isotropic properties; that is, it has similar elastic moduli in different directions. Second, with the increase in the volume fraction, the elastic modulus of the Gyroid structure gradually increases, and the change trend of the Zener anisotropy index is the same, indicating that it is increasingly isotropic, which is consistent with the results of other rigid lattice structures. In addition, we found that the elastic modulus of the Gyroid structural unit in the three directions [001], [100], and [010] was equal but lower than that in the other directions (Figure 6a). This means that its elastic modulus has an isotropic property in these three directions. Therefore, we select the elastic modulus of these three directions for nonlinear fitting, and the mathematical relationship is shown as follows:

(15) $$F{\left(T,V\right)}_{Gyroid}:T=1.154V+0.0126,$$

(16) $$F{\left(V,E\right)}_{Gyroid}E=11.96V^{1.9}+0.35,$$

To investigate the effect of 3D-printing layer anisotropy on the compression response, the layer orientation was studied. Due to the greater challenge of printing thin walls, a Gyroid structure with a constant volume fraction of 20% was chosen, which had the greatest possibility of anisotropy. In Section 2.3, the 3D process was introduced, and three samples (Figure 1) were prepared via printing under the participants. Compression tests were carried out in the X, Y, and Z directions (i.e., the [100], [010], and [001] directions, respectively). The results showed that the elastic modulus showed an obvious anisotropy trend in the horizontal direction. Compared with the layer orientation in the Z direction (i.e., [001] direction, 3D-printing construction direction), the response in the X and Y directions ([100] and [010] directions, perpendicular to the construction direction) is slightly lower, as shown in Table 3. In addition, we compared the test results with the nonlinear fitting mathematical relationship of the elastic modulus and found that the error of the three-dimensional elastic modulus simulated using the homogenization theory was small, with a maximum error of 22%, which could be used to predict the elastic modulus of the three-period minimal surface (Figure 6b).

### 3.2. Collaborative Optimization Design of Process and Structural Parameters

**Figure 7:**
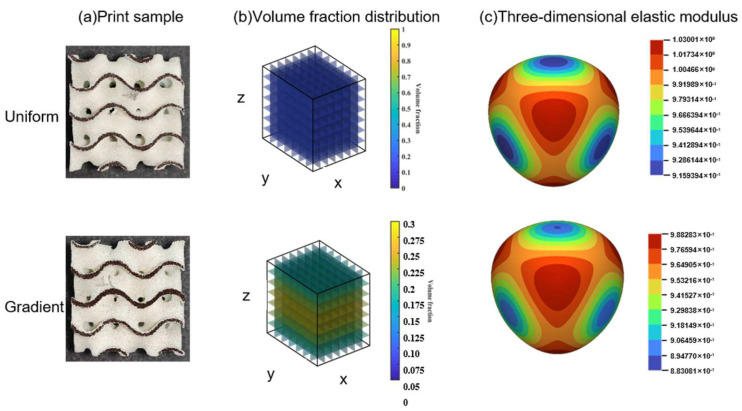
Uniform and gradient Gyroid structure SLS sample preparation. (**a**) Comparison of uniform and gradient structure printed samples. (**b**) Comparison of the variation in the distribution of integrals in the Z direction between uniform and gradient structures (**c**).Three-dimensional modulus surfaces of uniform structure and gradient structure.

According to the design method in Section 2.2, we designed the gradient Gyroid with an average volume fraction of 20%. First, in Section 3.1, we have learned the elastic anisotropy in the X, Y, and Z directions of the Gyroid structure with a 3D-printed constant volume fraction of 20%. These characteristics are affected by the SLS process parameters because the results of the homogenization theoretical simulation show that the elastic modulus of the Gyroid is isotropic in the X, Y, and Z directions (Figure 5). Therefore, we bring ΔE ≈ 0.2 obtained from Equation 3 into Equation 5 to determine ΔV = 2.5%, so our gradient Gyroid structure has a maximum volume fraction of 22.5% and a minimum volume fraction of 17.5%. From F(T, V) (Equation 15) we determine that the corresponding T ± ΔT is 0.2426 ± Δ0.041. The dimension of the Gyroid cell is 20 mm, so the gradient function T(z) along the z direction is the first function of the crossing points (T − ΔT, 20) and (T + ΔT, 20). As shown in Figure 7, a gradient Gyroid lattice structure with an average volume fraction of 20% is prepared using the same process parameters.

**Figure 8:**
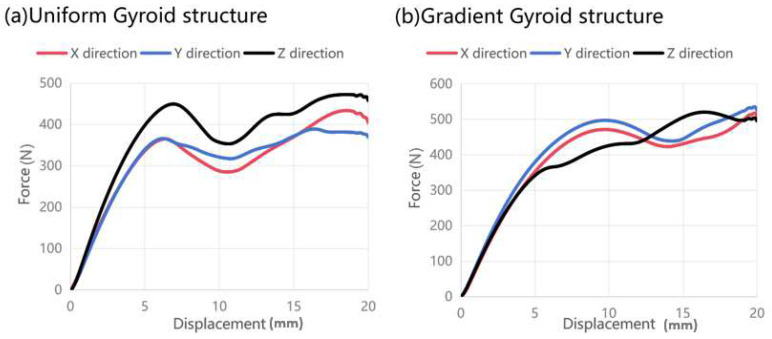
Compression load force and displacement curve. (**a**) Stress–strain curves of uniform lattice structure in x, y, and z directions. (**b**) Stress–strain curves of gradient lattice structure in x, y, and z directions.

To verify the effect of the gradient design on the optimization of 3D-printing anisotropy, compression tests were carried out. The force and displacement curves of a uniform Gyroid with a constant volume fraction of 20% and a gradient Gyroid with an average volume fraction of 20% are shown in Figure 8. First, in the elastic stage (0–5 mm displacement), the elastic modulus of the gradient Gyroid structure is approximately isotropic in the x, y, and z directions (Figure 8b), while the uniform Gyroid structure is obviously anisotropic (Figure 8a). Second, when the displacement of the two lattice structures exceeds 3 mm, they both show 3D-printing anisotropy, but the specific performance is different. For the uniform Gyroid structure, the force in the z direction is greater than that in the other directions. For a gradient Gyroid structure, the forces in the z direction are temporarily lower than those in the other directions. After further displacement is applied to 13 mm, the force in the z direction is greater than that in the other directions.

### 3.3. Functional Analysis of Gradient Lattice Structure

**Figure 9:**
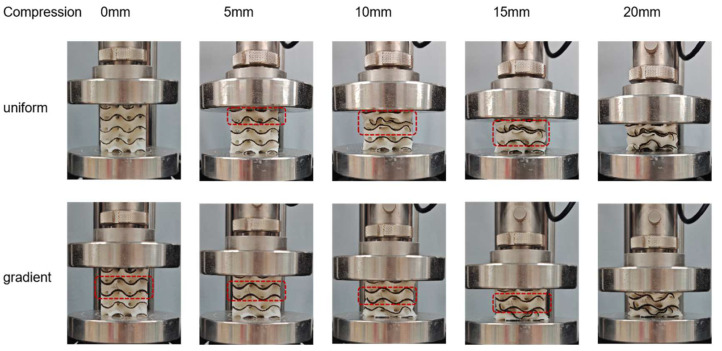
Compression test for loading in the z direction.

To further explore the mechanism and mechanical response of the gradient design to the anisotropic regulation of 3D-printed lattice structures, we analyzed the compression deformation behavior in the X, Y, and Z directions. First, for the homogeneous Gyroid structure, we observed the phenomenon of collapse by layer (Figure 9). During initial loading, the top layer cell wall of the structure begins to bend and produce elastic deformation. With the displacement gradually increasing to 10 mm, the pores inside the cell wall of the lattice structure gradually collapse, and the relative density of the structure increases. Finally, when the displacement is loaded to 20 mm, all the hole walls have basically collapsed. The gradient Gyroid structure in the compression process needs to be discussed in two cases. When the loading direction of the force is perpendicular to the direction of the volume fraction gradient (the x–y plane), that is, along the X and Y directions, the deformation is like that of the uniform Gyroid structure, but the overall stress level is higher than that of the uniform Gyroid structure (Figure 8). When the loading direction of the force is parallel to the direction of the volume fraction gradient (Figure 9), that is, along the Z direction, we observe that the deformation begins in the region with the smallest volume fraction distribution in the Z direction and then begins to collapse layer by layer along the direction where the volume fraction increases (in the middle). This phenomenon can be seen in the force-displacement curve of the gradient Gyroid structure (Figure 8b). When the displacement is less than 13 mm, the z-direction force response is less than that in the other directions, and the part corresponding to this minimum volume fraction begins to deform at this stage. After the displacement increases, the force response in the z direction is greater than that in the other directions, and this stage corresponds to the expansion of the deformation along the direction of the volume fraction increase. At the same time, we can also find that, compared with the force and displacement response curves of the uniform Gyroid structure, the shape of the force and displacement response curve of the gradient Gyroid structure in the z direction is not the same as that in the other directions, showing a gradual climbing phenomenon (5–15 mm displacement moment), and there is no obvious force decline after the end of the online elastic stage (5–10 mm).

**Figure 10:**
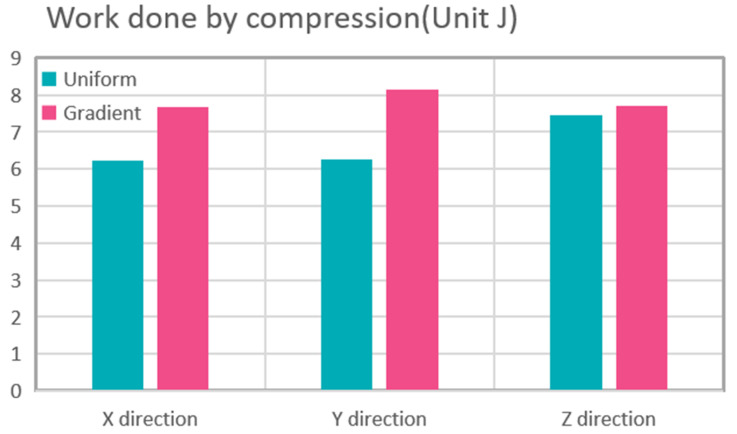
Work done by compression.

**Figure 11:**
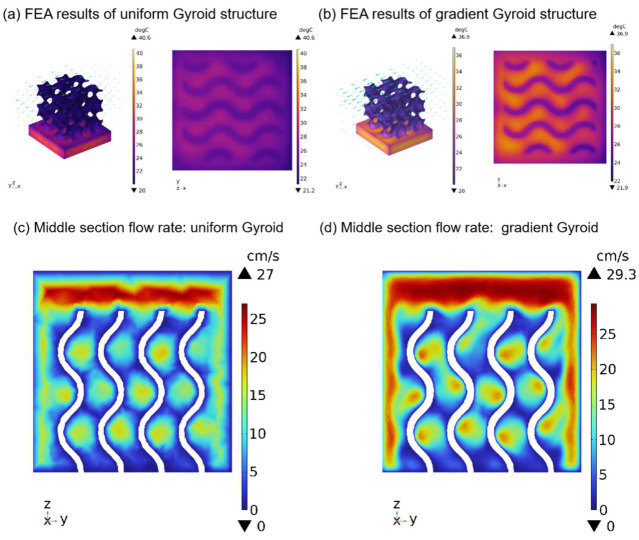
Finite element heat dissipation simulation.

Finally, we also discuss the function of the gradient structure to broaden its engineering application. First, we calculate the work performed by the force in the X, Y, and Z directions in the above compression tests to evaluate the energy absorption properties of the two structures. Second, we simulated the heat dissipation performance at room temperature. A heat source was placed under the uniform Gyroid structure and the gradient Gyroid structure. The heat consumption rate of the heat source was 0.5 W, the surface radiation emissivity to the thermal environment was 0.8, and the flow rate of air at room temperature (293.75 K) was 10 cm/s. In terms of absorption capacity, as shown in Figure 10, the energy absorption of the uniform structure and gradient structure in the x, y, and z directions is 6.227, 6.268, and 7.443 J and 7.66, 8.132, and 7.709 J. The gradient structure improves by 23.1%, 29.7%, and 4% in the X, Y, and Z directions, respectively. This shows that the energy absorption characteristics can be improved using the gradient structure design without increasing the mass of the lattice structure. In terms of heat dissipation, the gradient structure also has excellent performance. Given the geometry of the Gyroid, we apply the air fluid from the X direction, as this direction is most conducive to air flow. As a result (Figure 11a,b), the maximum surface temperature of the uniform and gradient structures is 40.6 °C and 36.9 °C, respectively, and the surface temperature decreases by 9.1% compared to the uniform Gyroid structure. At the same time, we calculated the air flow velocity of the middle section of the two structures along the Z direction (gradient direction) using finite element simulation (Figure 11c,d) and found that the maximum flow velocity of the uniform structure and the gradient structure was 27 cm/s and 29.3 cm/s, respectively, which indicated that the heat dissipation advantage of the gradient structure may have come from the fact that the uneven section of the gradient structure was more conducive to air flow and thus carried away more heat.

## 4. Discussion

The Gyroid structure, as a mechanical metamaterial, has approximately elastic isotropy and the same elastic modulus in the X, Y, and Z directions. The relationship between the curvature parameter T, the volume fraction V, and the elastic modulus fitted using linear and nonlinear simulations has a maximum error of 22%. The changing trend of the elastic modulus can be predicted to some extent.

The anisotropy of the 3D-printing process affects the anisotropy of the Gyroid structure, which is characterized by a higher elastic modulus in the Z direction than in the other directions. Using the process data- and equation-driven volume fraction gradient design method, the influence of the 3D-printing process on the elastic modulus of the isotropic structure can be effectively regulated so that it is approximately isotropic in the X, Y, and Z directions.

The volume fraction gradient design will affect the structure’s force and displacement curve and compression deformation mode. When the loading direction of the force is parallel to the direction of the volume fraction gradient, the deformation spreads along the gradient direction to the direction with the largest volume fraction. At the same time, the force and displacement curves are different from other directions and shapes, showing a gradual rise, and there is no obvious decline in the force value at the end of the online elastic stage, which indicates that the gradient structure is more stable.

On the premise of not changing the mass and volume of the structure, the energy absorption characteristics of the gradient structure in the X, Y, and Z directions are increased by 23.1%, 29.7%, and 4%, respectively, compared with those of the uniform structure. In terms of thermal performance, the air enters the porous heat dissipation structure perpendicular to the gradient direction. The gradient structure design decreased the temperature by 9.1% compared to the uniform Gyroid structure.

## 5. Conclusions

**Figure 12:**
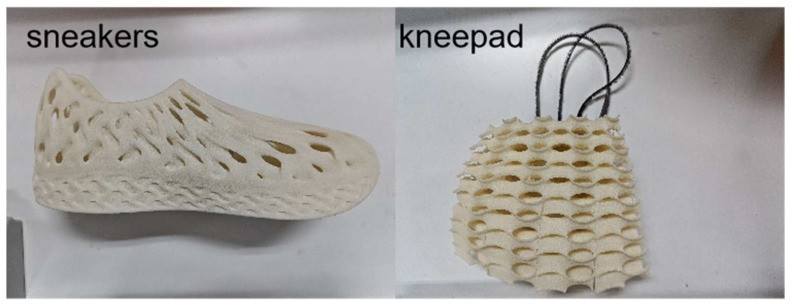
3D-printed flexible TPMS structural sports equipment.

This work aims to propose an optimal design technique based on process data for the geometric parameters of TPMS structures. The anisotropic effects introduced by the additive manufacturing process during the 3D printing of isotropic lattice structures can be effectively mitigated by adjusting the gradient design direction of the overall integration. Furthermore, via compression tests conducted in various directions, we elucidate the relationship between the gradient design direction of the volume fraction gradient and the direction of compression loading. Additionally, we clarify the role of the gradient design in controlling the mechanical characteristics of lattice structure filling parts via deformation modes. Sports equipment is one of the main engineering applications of flexible mechanical metamaterials, so we discuss the energy absorption characteristics of the flexible Gyroid structure and analyze the advantages of the gradient structure compared with the uniform structure in heat transfer using finite element heat transfer simulation. We explore the impact of the gradient design on the functioning of the lattice structure. The findings suggest that the volume fractional gradient design of TPMS structures provides added advantages in terms of heat dissipation, energy absorption, and other functionalities. This expands the potential applications of flexible TPMS lattice structures in sports equipment (Figure 12).

## Figures and Tables

**Table 1:** SLS parameters used in the production of lattice structures for mechanical testing.

Process Parameter	Value
Laser power	20 w
Laser scan speed	2500 mm/s
Laser hatch spacing	0.1 mm
Powder deposition thickness	0.15 mm

**Table 2:** Quality characterization of printed samples with uniform and gradient structures.

Type		Weight (g)	Dimension (X × Y × Z mm)
Gradient Gyroid structure	Sample 1	14.31	39.38 × 39.44 × 40.51
	Sample 2	13.57	39.33 × 39.55 × 40.50
	Sample 3	13.18	39.64 × 39.89 × 40.32
Uniform Gyroid structure	Sample 1	13.78	39.32 × 39.22 × 40.12
	Sample 2	14.26	39.53 × 39.23 × 40.48
	Sample 3	13.54	39.25 × 39.64 × 40.33

**Table 3:** SLS produces elastic modulus of uniform and gradient lattice structures.

Uniform Gyroid			Gradient Gyroid		
Modulus of Elasticity E (MPa)			Modulus of Elasticity E (MPa)		
X([100])	Y([010])	Z([001])	X([100])	Y([010])	Z([001])
0.988	0.975	1.175	1.001	1.106	1.025

## Data Availability

The data presented in this study are available on request from the corresponding author.

## References

[1] Álvarez-Trejo A., Cuan-Urquizo E., Bhate D., Roman-Flores A. (2023). Mechanical Metamaterials with Topologies Based on Curved Elements: An Overview of Design, Additive Manufacturing and Mechanical Properties. Mater. Des..

[2] Teawdeswan L., Dong G. (2023). Inverse Design of Multi-Material Gyroid Structures Made by Additive Manufacturing. Int. J. Mech. Sci..

[3] Tilton M., Borjali A., Griffis J.C., Varadarajan K.M., Manogharan G.P. (2023). Fatigue Properties of Ti-6Al-4V TPMS Scaffolds Fabricated via Laser Powder Bed Fusion. Manuf. Lett..

[4] Black S., Tzagiollari A., Mondal S., Dunne N., MacManus D.B. (2023). Mechanical Behaviour of Gel-Filled Additively-Manufactured Lattice Structures under Quasi-Static Compressive Loading. Mater. Today Commun..

[5] Jiang W., Yin G., Xie L., Yin M. (2022). Multifunctional 3D Lattice Metamaterials for Vibration Mitigation and Energy Absorption. Int. J. Mech. Sci..

[6] Berger J.B., Wadley H.N.G., McMeeking R.M. (2017). Mechanical Metamaterials at the Theoretical Limit of Isotropic Elastic Stiffness. Nature.

[7] Novak N., Tanaka S., Hokamoto K., Mauko A., Yilmaz Y.E., Al-Ketan O., Vesenjak M., Ren Z. (2023). High Strain Rate Mechanical Behaviour of Uniform and Hybrid Metallic TPMS Cellular Structures. Thin-Walled Struct..

[8] Liu Z., Gong H., Gao J., Liu L. (2023). Bio-Inspired Design, Mechanical and Mass-Transport Characterizations of Orthotropic TPMS-Based Scaffold. Compos. Struct..

[9] Lal Lazar P.J., Subramanian J., Natarajan E., Markandan K., Ramesh S. (2023). Anisotropic Structure-Property Relations of FDM Printed Short Glass Fiber Reinforced Polyamide TPMS Structures under Quasi-Static Compression. J. Mater. Res. Technol..

[10] Viswanath A., Khan K.A., Barsoum I. (2022). Design of Novel Isosurface Strut-Based Lattice Structures: Effective Stiffness, Strength, Anisotropy and Fatigue Properties. Mater. Des..

[11] Design for Additive Manufacturing of Functionally Graded Lattice Structures: A Design Method with Process Induced Anisotropy Consideration|SpringerLink. https://link.springer.com/article/10.1007/s40684-019-00173-7.

[12] Clough E.C., Plaisted T.A., Eckel Z.C., Cante K., Hundley J.M., Schaedler T.A. (2019). Elastomeric Microlattice Impact Attenuators. Matter.

[13] Feng H., Wang W., Wang T., Zhang L., Li W., Hou J., Chen S. (2023). Preparation of Dynamic Polyurethane Networks with UV-Triggered Photothermal Self-Healing Properties Based on Hydrogen and Ion Bonds for Antibacterial Applications. J. Mater. Sci. Technol..

[14] Yao D., Zhao Z., Wei Y., Li J. (2023). Gradient Scaffolds Developed by Parametric Modeling with Selective Laser Sintering. Int. J. Mech. Sci..

[15] Sala R. (2022). Insights into the Printing Parameters and Characterization of Thermoplastic Polyurethane Soft Triply Periodic Minimal Surface and Honeycomb Lattices for Broadening Material Extrusion Applicability. Addit. Manuf..

[16] Prajapati M.J., Kumar A., Lin S.-C., Jeng J.-Y. (2022). Multi-Material Additive Manufacturing with Lightweight Closed-Cell Foam-Filled Lattice Structures for Enhanced Mechanical and Functional Properties. Addit. Manuf..

[17] Maskery I., Aremu A.O., Parry L., Wildman R.D., Tuck C.J., Ashcroft I.A. (2018). Effective Design and Simulation of Surface-Based Lattice Structures Featuring Volume Fraction and Cell Type Grading. Mater. Des..

[18] Ren F., Zhang C., Liao W., Liu T., Li D., Shi X., Jiang W., Wang C., Qi J., Chen Y. (2021). Transition Boundaries and Stiffness Optimal Design for Multi-TPMS Lattices. Mater. Des..

[19] Feng J., Liu B., Lin Z., Fu J. (2021). Isotropic Porous Structure Design Methods Based on Triply Periodic Minimal Surfaces. Mater. Des..

[20] Xu Z., Ha C.S., Kadam R., Lindahl J., Kim S., Wu H.F., Kunc V., Zheng X. (2020). Additive Manufacturing of Two-Phase Lightweight, Stiff and High Damping Carbon Fiber Reinforced Polymer Microlattices. Addit. Manuf..

[21] Han W., Kong L., Xu M. (2022). Advances in Selective Laser Sintering of Polymers. Int. J. Extrem. Manuf..

[22] Fan J., Zhang L., Wei S., Zhang Z., Choi S.-K., Song B., Shi Y. (2021). A Review of Additive Manufacturing of Metamaterials and Developing Trends. Mater. Today.

[23] Yuan S., Li S., Zhu J., Tang Y. (2021). Additive Manufacturing of Polymeric Composites from Material Processing to Structural Design. Compos. Part B Eng..

[24] Martin D.J., Osman A.F., Andriani Y., Edwards G.A. (2012). Thermoplastic Polyurethane (TPU)-Based Polymer Nanocomposites. Advances in Polymer Nanocomposites.

[25] Rajitha K., Mohana K.N.S., Hegde M.B., Nayak S.R., Swamy N.K. (2020). Fabrication of ZnO/rGO and ZnO/MWCNT Nanohybrids to Reinforce the Anticorrosion Performance of Polyurethane Coating. FlatChem.

[26] Zhao Z., Wu Z., Yao D., Wei Y., Li J. (2023). Mechanical Properties and Failure Mechanisms of Polyamide 12 Gradient Scaffolds Developed with Selective Laser Sintering. J. Mech. Behav. Biomed. Mater..

[27] Arefin A.M.E., Khatri N.R., Kulkarni N., Egan P.F. (2021). Polymer 3D Printing Review: Materials, Process, and Design Strategies for Medical Applications. Polymers.

[28] Lin X., Hao M., Ying J., Wang R., Lu Y., Gong M., Zhang L., Wang D., Zhang L. (2022). An Insight into the Tensile Anisotropy of 3D-Printed Thermoplastic Polyurethane. Addit. Manuf..

[29] Dadbakhsh S., Verbelen L., Vandeputte T., Strobbe D., Van Puyvelde P., Kruth J.-P. (2016). Effect of Powder Size and Shape on the SLS Processability and Mechanical Properties of a TPU Elastomer. Phys. Procedia.

[30] Xu T., Shen W., Lin X., Xie Y.M. (2020). Mechanical Properties of Additively Manufactured Thermoplastic Polyurethane (TPU) Material Affected by Various Processing Parameters. Polymers.

[31] Holmes D.W. (2022). Mechanical behaviour of flexible 3D printed gyroid structures as a tuneable replacement for soft padding foa. Addit. Manuf..

[32] Maskery I., Ashcroft I.A. (2020). The Deformation and Elastic Anisotropy of a New Gyroid-Based Honeycomb Made by Laser Sintering. Addit. Manuf..

[33] Maskery I., Parry L.A., Padrão D., Hague R.J.M., Ashcroft I.A. (2022). FLatt Pack: A Research-Focussed Lattice Design Program. Addit. Manuf..

